# Sex differences in the impact of social determinants of health on substance use disorder treatment outcomes

**DOI:** 10.1186/s13293-025-00734-3

**Published:** 2025-07-22

**Authors:** C. Leonardo Jimenez Chavez, MacKenzie R. Peltier, Sherry A. McKee

**Affiliations:** 1 Department of Psychiatry, Yale School of Medicine, 40 Temple Street, Suite 5B, New Haven, CT 06510 USA; 2 VA Connecticut Healthcare System, Mental Health Service, West Haven, CT USA; 3 Clinical Neuroscience Division, National Center of PTSD, West Haven, CT USA

**Keywords:** Sex differences, Social determinants of health, Substance use disorder, Treatment outcomes, Community Treatment

## Abstract

**Background:**

Social determinants of health (SDOH) and clinical severity factors are known to shape substance use disorder (SUD) treatment outcomes, yet limited research has explored how these influences differ by sex. Understanding these differences is important to improving treatment equity and outcomes in publicly funded treatment systems.

**Methods:**

This study analyzed data from the 2018–2022 Treatment Episode Data Set-Discharges (TEDS-D), a national dataset of adults discharged from publicly funded SUD treatment programs. Sex-stratified binary logistic regressions were used to examine predictors of two outcomes: treatment non-completion and substance use at discharge. Predictors included SDOH (i.e., employment, education level, housing status, criminal justice involvement, prior treatment history, marital status, health insurance coverage and treatment duration) and indicators of SUD severity (e.g., age at first use, polysubstance use, and co-occurring psychiatric disorders).

**Results:**

Both SDOH and clinical severity indicators were significantly associated with poorer treatment outcomes, with distinct patterns by sex. Women showed more consistent risk for poor treatment outcomes across predictors, including unemployment, psychiatric comorbidities, and polysubstance use, while lack of prior treatment history was the strongest predictor of substance use at discharge and dropout for men. Other predictors, such as housing instability, criminal justice involvement, and later-onset substance use, were also associated with increased risk of non-abstinence and dropout, with notable sex differences. Health insurance coverage was associated with better outcomes for both sexes, with the protective effect more consistent in women.

**Conclusions:**

These findings emphasize the need for sex-informed treatment approaches that address both social determinants of health and clinical complexity. Tailoring care to the unique risks and contexts of men and women may improve retention and reduce substance use at discharge, particularly in publicly funded systems.

**Highlights**

- We examined social determinants of health (SDOH), and substance use disorder (SUD) severity-related predictors of substance use and treatment completion in a national sample of approximately 7 million adults.
- Women demonstrated more consistent vulnerability across predictors, including unemployment, co-occurring psychiatric disorders, and polysubstance use.
- For men, lack of prior treatment for SUD was the most consistent predictor for substance use at discharge and treatment dropout.
- Housing instability, access to healthcare, and financial barriers showed sex-specific effects, with women generally experiencing great risk of unsuccessful treatment.
- Findings highlight the importance of improving SUD care to address sex-specific risks and structural barriers, especially in publicly funded systems.

**Plain English Summary**

Substance use treatment is not a one-size-fits-all process. Recovery is shaped by both structural challenges, such as housing instability or limited access to care, and the clinical severity of substance use. These factors influence whether someone completes treatment and stays abstinent, and they often affect men and women in different ways. In this study, we analyzed data from approximately 7 million publicly funded substance use treatment episodes across the United States. We looked at how social determinants of health (e.g. employment status, education, housing, access to treatment) and clinical factors (e.g. age of substance use onset, psychiatric comorbidities and polysubstance use), were associated with two key outcomes: whether a person completed treatment and whether they reported use of their primary substance at the end of care. We found that women often faced greater challenges, especially regarding unemployment, co-occurring mental health conditions and using more than one type of substance. For men, being new to treatment was a strong predictor of poorer treatment success. These findings demonstrate the need for treatment programs to offer support that meets men and women where they are, considering the different barriers and challenges each group may face along the path to sustained recovery.

## Background

Substance Use Disorders (SUDs) are a major global public health concern, contributing to significant health and social burdens that affect millions of individuals, their families and their communities. In the United States, estimates from the Substance Abuse and Mental Health Services Administration (SAMHSA) approximate that nearly 18% of the population had an SUD diagnosis in 2023 [34]. While treatment improves SUD outcomes, 76% of individuals with SUD did not receive treatment [34]. There are numerous barriers to treatment which are influenced by both individual and structural factors, including social determinants of health (SDOH).

SDOH are nonmedical conditions in which people are born, grow, live, work, worship and age that influence a wide range of health and quality-of-life outcomes and risks (Centers for Disease Control and Prevention [CDC], [5]). These factors include employment, education, housing stability, healthcare access, and criminal justice involvement. Studies have demonstrated improved SUD treatment outcomes, including increased retention and reduced substance use, when interventions reduce social stressors and provide recovery support [2, 27]. Employment status and social support, for example, are strong predictors of better retention in treatment and long-term abstinence [18]. Conversely, individuals who face unemployment, homelessness, or social isolation exhibit a greater risk of non-abstinence [18]. Involvement with the criminal justice system is also associated with worse treatment outcomes, disrupting treatment continuity and disproportionately overwhelming marginalized groups [26]. The influence of SDOH is particularly evident in publicly funded SUD treatment programs, which often serve the most socioeconomically disadvantaged populations. Individuals in these programs frequently face financial instability, lack of reliable transportation, and limited healthcare access, all factors that hinder treatment retention [1]. Efforts to address these social challenges have demonstrated promise in improving engagement in treatment programs and reducing post-treatment drug use [6, 29].

Sex differences further influence how SDOH play a role in SUD treatment outcomes. Women often face compounding structural and social barriers, including childcare responsibilities, financial dependence, intimate partner violence and stigma, that all impact women’s ability to remain engaged in treatment [13, 19, 22]. These challenges not only impede treatment continuity but can also interfere with the overall progress to abstinence. Conversely, men are more likely to be involved in the legal system and have higher rates of polysubstance use, factors that shape both access to and outcomes of treatment [12, 32]. Additionally, employment and economic stability play a larger role in treatment retention for men, as job loss or financial instability is a consistent reason for treatment discontinuity [24]. Despite these well-documented sex differences, research examining the interaction between SDOH and sex in shaping treatment trajectories remains limited.

In addition to SDOH, substance use severity characteristics also significantly impact treatment outcomes. Variables including age at first use, psychiatric comorbidities and polysubstance use, serve as important predictors of treatment success. An earlier age of first use is associated with increased SUD severity and poorer treatment outcomes [8]. Psychiatric comorbidities further complicate treatment, as individuals with existing dual diagnoses are reported to have a greater likelihood of continued use and treatment non-completion [17], with women in particular showing higher rates of co-occurring conditions, which further complicate treatment adherence and delay recovery [19]. Polysubstance use also presents additional treatment challenges, often requiring more robust and long-term interventions [3]. While these severity-related factors play a role in influencing treatment outcomes, their interplay with SDOH and interaction with sex remains unclear, thus highlighting the need for a large-scale, data-driven approach to investigate the influence of both SDOH and SUD severity-related factors of treatment outcomes across sexes.

To address this gap in the literature, the present study uses data from the national Treatment Episode Data Set-Discharges (TEDS-D), a dataset of annual SUD treatment discharges reported by SAMHSA. TEDS-D provides extensive information on patient demographics, social and economic indicators, substance use history, medical and treatment history and discharge status from individuals discharged from publicly funded SUD treatment programs across the United States. Using this comprehensive dataset, we investigated how SDOH (i.e., employment, education, housing, criminal justice involvement, marital status, prior treatment history, health insurance coverage and treatment duration) and SUD severity-related factors (i.e., age of first use, polysubstance use, and presence of a co-occurring psychiatric disorder) influence SUD treatment success and substance use outcomes and whether these relationships differ between men and women. We hypothesized that key SDOH and SUD severity-related factors, reflecting known structural and clinical barriers, would significantly predict poorer treatment outcomes, and that these predictive associations would be stronger in women given their amplified vulnerabilities to these risk factors.

## Materials and methods

### Data source and sample

The present analysis included all TEDS-D records from 2018 to 2022. This five-year period rendered a large sample size and allowed for the study of current trends in SUD treatment completion and substance use at discharge.

### Treatment outcomes

Two primary outcome variables were analyzed: (1) substance use at discharge (non-abstinent vs. abstinent) and (2) treatment completion (non-completion vs. completion).

*Substance Use at Discharge* was determined based on the primary substance recorded at discharge, as reported by treatment facilities at client discharge. Individuals who did not report using any substance at discharge were categorized as abstinent, while those who had a recorded substance use at discharge were categorized as non-abstinent. Episodes with missing substance use data at discharge were excluded.

*Treatment Completion* was determined based on an individual’s discharge status. The discharge “reason” variable in the dataset was recoded into a binary outcome variable, where individuals who successfully completed treatment were classified as treatment completers, while those who did not complete treatment for any reason (e.g., dropped out, transferred, administrative discharge) were classified as non-completers. Episodes where the discharge reason was recorded as “death” were excluded from the analysis.

### Predictor variables

A range of independent variables were included to examine their influence on treatment completion and substance use at discharge.

#### SDOH variables

Variable selection was guided by the Health People 2030 social SDOH framework, which identifies five key domains influencing health outcomes: economic stability, education access and quality, health care access and quality, neighborhood and built environment and social and community context (Office of Disease Prevention and Health Promotion [ODPHP], [21]). In this study, core SDOH predictors included employment status (employed vs. unemployed), education level (categorized as high school or less vs. some college or more), housing status (housed vs. unhoused), and criminal justice involvement (history of prior arrests vs. none). Marital status (married vs. not married) was included as an indicator of social support, reflecting the social and community context domain. To capture healthcare access and quality, we included prior treatment history (had prior treatment vs. no prior treatment), health insurance type (none vs. public vs. private insurance coverage), and treatment duration (<30 days vs. 31+ days).

#### SUD severity-related variables

Key SUD severity-related predictors included age of first substance use (17 years or younger, 18–29 years, or 30+ years), as well as a polysubstance use score, calculated by summing the substance-specific variables available in the dataset. These variables denote whether a specific substance (e.g. alcohol, cocaine, methamphetamine, etc.) was reported at admission, with each substance coded as present or not. A higher score indicates a greater number of substances used and increased substance use severity. We also included a binary predictor indicating the presence or absence of a co-occurring psychiatric disorder at admission (mental health diagnosis vs. none).

#### Other covariates

Demographic variables including age (young adults: 18–29 years, middle-aged adults: 30–49 years, older adults: 50+ years), sex (men, women), race (White, Black/African American, American Indian/Alaska Native or Asian/Pacific Islander, and Other), and ethnicity (Hispanic/Latino, Not Hispanic/Latino) were included as covariates.

### Sample selection and data cleaning

To maintain consistency in subgroup analyses, our sample was limited to adults (ages 18 and older) and episodes with missing demographic data (race, sex, or ethnicity) were excluded. Additionally, individuals whose primary substance at admission was listed as 'none' were excluded (11.49%) and the study focused on individuals with reported, current substance use at admission. For all predictor variables, episodes with missing data were excluded using listwise deletion at the model level. The final sample included a total of 7,522,532 adults (36% women) presenting with SUD.

### Statistical analysis

All variables included in the analysis were categorical. To examine the influence of SDOH and SUD severity-related variables on treatment completion and substance use at discharge, a series of binary logistic regression models were conducted. First, individual binary logistic regression models were run for each SDOH and SUD severity-related predictor individually, while controlling for covariates. Each model included age, race, and ethnicity as covariates to adjust for potential confounding effects. To evaluate whether the relationship between each predictor and the outcome variables differed by sex, main effect and interaction terms (sex × predictor) were included in the models. If a significant sex × predictor interaction was detected (*p* < 0.05), stratified analyses were conducted by running the same logistic regression models separately for men and women to examine sex-specific effects. Following the individual predictor analyses, sex-stratified multivariate binary logistic regression models were conducted separately for men and women. Each model included all SDOH and SUD predictors, along with covariates. A backward Wald method was applied to systematically remove non-significant variables and identify the strongest model for predicting treatment completion and substance use at discharge. All significant predictors from prior analysis continued to remain significant in the final sex-stratified multivariate logistic regression models for substance use at discharge and treatment non-completion. Thus, we have not included the multivariate models as they did not provide any new information. All analyses were conducted using SPSS (Version 29) and JMP Pro (Version 17.2). Results were reported as odds ratios (OR) with 95% confidence intervals (CI), and statistical significance was set at *p* < 0.05. Bonferroni corrections for multiple comparisons did not alter the pattern of findings (i.e., all significant findings remained significant).

## Results

Descriptive statistics for all covariates, predictors, and treatment outcome variables are shown in Table 1, providing an overview of the sample characteristics and variables examined in the study.

**Table 1:** Percentage distributions for covariates, predictors, and outcome variables (*N* = 7,526,148)

Variable		Subcategory	Percent of total
Demographics	Sex	Males	64.43%
		Females	35.57%
	Age	Young adults	44.47%
		Middle-aged adults	53.93%
		Older adults	1.60%
	Race	White	68.32%
		Black/African American	18.05%
		AIAN/API	4.39%
		Other	9.24%
	Ethnicity	Hispanic/Latino	13.34%
		Not Hispanic/Latino	86.66%
Social Determinants of Health	Employment	Employed	28.48%
		Unemployed	71.52%
	Education	High school or less	74.02%
		Some college or more	25.98%
	Housing	Housed	86.98%
		Unhoused	13.02%
	Recent arrests	None	94.09%
		Yes	5.91%
	Marital status	Married	12.66%
		Not married	87.34%
	*Access to Treatment Variables:*		
	Prior treatment	Had treatment	61.32%
		No prior treatment	38.68%
	Health insurance type	None	27.95%
		Private	8.37%
		Public/Government	63.68%
	Treatment duration	<30 days	54.26%
		31+ days	45.74%
SUD Severity	Age at first use	<17 years	47.51%
		18 to 29 years	40.00%
		30+ years	12.50%
	Co-occurring disorders	No	54.44%
		Yes	45.56%
	Polysubstance use	1 substance	42.79%
		2 substances	34.82%
		3 substances	22.39%
Outcomes	Discharge status	Use at discharge	90.85%
		Abstinent	9.15%
	Treatment completion	Yes	42.85%
		No	57.15%

### Substance use at discharge

*SDOH.* As shown in Table 2, several SDOH were significantly associated with substance use at discharge. Risk of continued use upon discharge was higher among individuals who were unemployed, had attained some college education or higher, had a recent arrest history, or were entering treatment for the first time. In contrast, having either public or private health insurance coverage, compared to no insurance, was associated with lower risk of substance use at discharge. While these patterns were generally consistent across sexes, some differences in the strength of the associations were observed. Unemployment and recent arrests were linked to increased risk of continued substance use at discharge for both men and women, with effects more pronounced in women. Higher education levels and lack of prior treatment were also associated with increased risk of substance use upon discharge in both sexes but showed stronger effects in men. Health insurance coverage was protective across sexes, with a slightly stronger protective effect in women. Housing status, marital status, and treatment duration were not significantly associated with substance use at discharge in either sex.

**Table 2:** Odds ratios for predictors of substance use at discharge, stratified by sex

			Men	Women	
Predictor	Level	Overall interaction significance with sex	OR (95% CI)	OR (95% CI)	Men vs. Women Comparison OR (95% CI); Males = REF
*Social determinants of health*					
Employment	Employed	<0.001	REF	REF	
	Unemployed		1.13 (1.12, 1.14)^a^	1.25 (1.24, 1.27)^a^	1.08 (1.07, 1.10)^a^
Education	High school or less	<0.001	REF	REF	
	Some college or more		1.33 (1.32, 1.34)^a^	1.22 (1.21, 1.24)^a^	0.92 (0.90, 0.93)^a^
Housing	Housed	n.s			
	Unhoused				
Recent arrests	None	<0.001	REF	REF	
	Yes		1.23 (1.21, 1.26)^a^	1.27 (1.25, 1.30)^a^	1.05 (1.02, 1.08)^a^
Marital status	Married	n.s			
	Not married				
Prior treatment	Had treatment	<0.001	REF	REF	
	No prior treatment		1.31 (1.30, 1.32)^a^	1.22 (1.21, 1.23)^a^	0.94 (0.93, 0.95)^a^
Health insurance type	None	<0.001	REF	REF	
	Private		0.21 (0.21, 0.22)^a^	0.17 (0.16, 0.17)^a^	0.76 (0.74, 0.78)^a^
	Public/government		0.28 (0.28, 0.28)^a^	0.25 (0.24, 0.25)^a^	0.87 (0.85, 0.88)^a^
Treatment duration	<30 days	n.s			
	31+ days				
*SUD severity*					
Age at first use	<17 years	<0.001	REF	REF	
	18 to 29 years		0.82 (0.81, 0.83)^a^	0.94 (0.93, 0.95)^a^	1.15 (1.13, 1.16)^a^
	30 + years		0.88 (0.87, 0.89)^a^	1.05 (1.03, 1.07)^a^	1.15 (1.13, 1.18)^a^
Co-occurring psychiatric disorders	No	<0.001	REF	REF	
	Yes		0.96 (0.95, 0.96)^a^	1.20 (1.18, 1.21)^a^	1.25 (1.24, 1.27)^a^
Polysubstance use	1 substance	<0.001	REF	REF	
	2 substances		1.31 (1.30, 1.33)^a^	1.44 (1.42, 1.45)^a^	1.10 (1.08, 1.11)^a^
	3 substances		1.77 (1.75, 1.79)^a^	2.03 (2.00, 2.06)^a^	1.16 (1.13, 1.18)^a^

Note: Last column shows ORs for Predictor × Sex interaction (F vs. M; M = REF); ^a^*p* < 0.01; ^b^*p* > 0.01; *n.s.* = no significant difference

#### SUD severity-related predictors

Several indicators of clinical severity were associated with greater risk of substance use at discharge. Polysubstance use showed a dose–response relationship, with use of two or more substances increasing risk compared to single-substance use. Later age of first substance use was generally protective such that initiation at 18–29 or 30+ years of age was associated with lower odds of substance use at discharge compared to those who started before age 17. Co-occurring psychiatric disorders were also associated with elevated risk for substance use at discharge, though not uniformly. These associations varied by sex, where women showed greater vulnerability related to both polysubstance use and co-occurring psychiatric disorders, while the protective effects of later substance use onset were stronger and more consistent among men. Notably, substance use beginning after age 30 remained protective for men but associated with higher odds of substance use at discharge among women.

### Treatment non-completion

*SDOH.* As presented in Table 3, risk of treatment non-completion was higher among individuals who were unemployed, unhoused, unmarried, or entering treatment for the first time. Conversely, having health insurance, whether public or private, was associated with a lower likelihood of dropout. Risk for treatment attrition varied for certain predictors based on sex. Women showed greater risk of not completing treatment associated with unemployment and unstable housing, while men showed greater vulnerability for attrition associated with being unmarried, lacking prior treatment experience, and having a recent arrest. While the sex differences for marital status and prior treatment history were statistically significant, the magnitude of these effects was modest. In contrast, differences by health insurance type were more consistent across sexes. Health insurance coverage reduced dropout risk in both sexes, with public insurance coverage showing a slightly stronger effect among women and private coverage showing modestly stronger effect among men. Education level was not significantly associated with treatment non-completion in either sex.

**Table 3:** Odds ratios for predictors of treatment non-completion, stratified by sex

			Men	Women	
Predictor	Level	Overall interaction significance with sex	OR (95% CI)	OR (95% CI)	Men vs women comparison OR (95% CI); Males = REF
*Social determinants of health*					
Employment	Employed	<0.001	REF	REF	
	Unemployed		1.53 (1.53, 1.54)^a^	1.66 (1.65, 1.67)^a^	1.08 (1.07, 1.09)^a^
Education	High school or less	n.s			
	Some college or more				
Housing	Housed	<0.001	REF	REF	
	Unhoused		1.11 (1.11, 1.12)^a^	1.34 (1.33, 1.36)^a^	1.21 (1.19, 1.22)^a^
Recent arrests	None	<0.001	REF	REF	
	Yes		1.19 (1.18, 1.20)^a^	1.01 (1.00, 1.02)^b^	0.85 (0.84, 0.87)^a^
Marital status	Married	0.001	REF	REF	
	Not married		1.08 (1.07, 1.08)^a^	1.06 (1.05, 1.07)^a^	0.98 (0.97, 0.99)^a^
Prior treatment	Had treatment	<0.001	REF	REF	
	No previous treatment		1.16 (1.16, 1.17)^a^	1.15 (1.14, 1.15)^a^	0.99 (0.98, 0.99)^a^
Health insurance type	None	<0.001	REF	REF	
	Private		0.65 (0.64, 0.65)^a^	0.67 (0.66, 0.68)^a^	1.04 (1.02, 1.06)^a^
	Public/government		0.77 (0.77, 0.78)^a^	0.72 (0.72, 0.73)^a^	0.93 (0.92, 0.94)^a^
Treatment duration	<30 days	<0.001	REF	REF	
	31+ days		1.02 (1.01, 1.02)^a^	0.96 (0.95, 0.96)^a^	0.94 (0.94, 0.95)^a^
*SUD severity*					
Age at first use	<17 years	<0.001	REF	REF	
	18 to 29 years		1.18 (1.17, 1.18)^a^	1.24 (1.23, 1.24)^a^	1.05 (1.04, 1.05)^a^
	30+ years		1.59 (1.58, 1.60)^a^	1.52 (1.51, 1.53)^a^	0.96 (0.96, 0.99)^a^
Co-occurring Psychiatric disorders	No	<0.001	REF	REF	
	Yes		1.47 (1.47, 1.48)^a^	1.56 (1.55, 1.57)^a^	1.20 (1.20, 1.21)^a^
Polysubstance use	1 substance	<0.001	REF	REF	
	2 substances		1.26 (1.25, 1.27)^a^	1.29 (1.28, 1.30)^a^	1.02 (1.01, 1.03)^a^
	3 substances		1.41 (1.40, 1.42)^a^	1.53 (1.52, 1.54)^a^	1.08 (1.07, 1.09)^a^

Last column shows ORs for Predictor × Sex interaction (F vs. M; M = REF); ^a^*p* < .01; ^b^*p* > .01; *n.s.* = no significant difference

#### SUD severity-related predictors

Later onset of substance use, co-occurring psychiatric disorders, and polysubstance use were all associated with increased risk of treatment non-completion. Individuals who initiated substance use at age 30 or older had the highest likelihood of dropout, as did those reporting concurrent mental health diagnoses or using multiple substances at treatment admission. The strength of these associations varied by sex. Women showed greater risk related to psychiatric comorbidities and polysubstance use, while men exhibited greater risk when entering treatment after age 30. All results are reported in Table 3.

## Discussion

This study examined how SDOH and SUD severity-related factors influence treatment outcomes in publicly funded SUD programs, with a focus on sex differences. Unlike, earlier sex-specific SUD investigations that assessed single predictors in smaller clinical samples, we leveraged a large, national representative dataset to evaluate both social and clinical vulnerabilities. Overall, we found that both SDOH and SUD severity indicators were strongly associated with substance use at discharge and treatment non-completion. Social and severity-related factors, such as unemployment, psychiatric comorbidity, and polysubstance use were consistently associated with poorer outcomes, especially for women. In contrast, fewer predictors, such as the lack of prior treatment, were more strongly associated with worse treatment outcomes in men, especially for substance use at discharge. These findings suggest that sex plays an important role in shaping how social and clinical vulnerabilities influence treatment outcomes.

### SDOH and treatment outcomes

Economic instability significantly impacted both substance use at discharge and treatment completion, with women more vulnerable to these effects. Unemployment was a consistent predictor of worse treatment outcomes across sexes, but the impact was stronger in women in both treatment outcomes. While employment is generally protective against treatment dropout for both men and women, women may be more vulnerable to the effects of unemployment due to compounded stressors including financial strain and caregiving responsibilities [13, 22]. These stressors may interfere with treatment retention and increase the likelihood of continued use.

Similarly, educational attainment was protective against substance use at discharge; however, this also showed sex-differences in associations. Individuals with at least some college education had greater odds of substance use at discharge compared to those with a high school education or less, especially among men. Interestingly, this observation was in an unexpected direction given that higher education levels are typically associated with better health outcomes. However, this pattern may be reflective of heavier substance use among college-aged men, consistent with existing research [14]. Nevertheless, the dataset used did not include direct information on substance use severity, limiting our ability to determine whether this association reflects pre-treatment use patterns.

Housing instability was associated with a decreased risk for treatment completion. While housing status did not significantly predict substance use at discharge, it was strongly associated with elevated dropout risk, especially among women. These findings reinforce the importance of housing stability for women to remain active in treatment programs [16], especially since unhoused women often face compounding structural and interpersonal barriers to care despite reporting high motivation to complete treatment [35].

History of recent arrests also impacted treatment outcomes, with specific sex differences emerging. Arrest history predicted continued substance use at discharge in both sexes, though the effect was stronger in women. This pattern aligns with existing literature demonstrating that legal system involvement often co-occurs with trauma, caregiving stress, and family system instability [4, 31], factors that women are particularly sensitive to and thus may translate to poorer treatment outcomes in women. Additionally, recent arrests were more strongly associated with treatment dropout among men only. This is consistent with research that there is lower engagement in court-mandated care [11]. For women, results suggest that while they were more compliant with attending treatment, they were nonetheless more likely to be using substances at discharge.

Marital status, used as an indicator of social support was associated with a slightly increased risk of treatment non-completion among unmarried individuals, with a statistically stronger effect in men, though the magnitude of this sex difference was modest. While marital status may not always reflect the presence or quality of social support (e.g. a partner may be emotionally unavailable or unsupportive), it can still serve as a general indicator of access to close relational social support. This may reflect sex differences in the availability and reliance on emotional support, as men are more likely to depend on romantic partners for support than women, who have broader social support networks outside their relationships [36].

#### Access to treatment

Individuals entering treatment for the first time had higher odds of both treatment non-completion and substance use at discharge compared to those with prior treatment experience, with both outcomes more pronounced in men. However, the magnitude of the sex difference was small for treatment non-completion, while the effect on substance use at discharge was more substantial. These findings support the evidence that recovery is rarely linear, often requiring multiple episodes of care over time as individuals cycle through periods of substance use, treatment, and re-engagement [10]. Health insurance coverage was a consistent protective factor across both treatment outcomes, though the strength of these associations varied slightly by sex. Compared to individuals without insurance coverage, those with private or public coverage had lower odds of substance use at discharge and were more likely to complete treatment. However, this protective effect was more pronounced in women related to substance use at discharge, while men showed slightly stronger benefits from private coverage in reducing dropout. These patterns may reflect reduced financial burden and continuity of care, as consistent health insurance coverage has shown to improve utilization of substance use, mental health and related services, especially among low-income adults with SUDs [23]. These findings may reflect differences in treatment quality and consistency of care across the different funding sources, suggesting that access to well-funded care plans play an important role in supporting positive outcomes. Lastly, longer treatment duration was slightly protective against treatment non-completion, with stays of 31 days or more associated with reducing dropout risk in women. This pattern aligns with prior work showing that extended treatment is particularly beneficial in female-specific programs [9].

### SUD severity-related predictors of treatment outcomes

Later onset of substance use was associated with lower odds of substance use at discharge, particularly among men and women who initiated use between ages 18–29. This finding aligns with research showing that earlier initiation is associated with more severe and persistent substance use trajectories [15, 28]. In contrast, later age of substance use onset was associated with increased risk of treatment non-completion compared to early onset (<17 years old), with the strongest effects observed among individuals whose use began at the age of 30 or older, in both men and women. For men, this may reflect entry into treatment in response to external pressures, such as job- or legal-related consequences, rather than intrinsic motivation to change [37]. For women, life responsibilities, such as employment or family responsibilities, may interfere with their ability to remain in treatment over time [4, 13]. Notably, this pattern may also reflect a broader public health trend of increasing substance use among older women, often in response to stress, isolation, or changing social roles [25, 33].

Polysubstance use predicted both substance use at discharge and non-completion, especially in women, with outcomes worsening as the number of substances increased. Women who reported using three substances had the highest likelihood of continued use and dropout, consistent with prior findings that polysubstance-using women often present with more complex clinical profiles, including co-occurring psychiatric conditions and poorer physical health compared to men [20, 30].

Co-occurring psychiatric disorders were associated with poorer treatment outcomes, particularly among women. This is highly consistent with prior research showing that women with SUDs are more likely to experience conditions such as depression, anxiety, and posttraumatic stress disorder [7, 38], which can complicate recovery and increase vulnerability to continued use even after treatment ends.

#### Clinical implications

These findings emphasize the importance of tailoring SUD treatment approaches based on both SDOH and clinical severity, while accounting for sex-specific risk patterns. While several predictors, such as unemployment, psychiatric comorbidities and polysubstance use, were significant across sexes, women showed a more consistent pattern of vulnerability associated with poorer treatment outcomes. This suggests that more comprehensive treatment models, that incorporate trauma-informed care, stronger mental health support, and logistical support systems like childcare and transportation, may be especially beneficial for women. Among men, risk patterns varied more across predictors and treatment outcomes, but an absence of prior treatment stood out as a consistent predictor associated with both substance use at discharge and treatment dropout. Although the sex difference in dropout related to prior treatment history was modest, this finding emphasizes the need for stronger engagement strategies for men entering treatment for the first time. Programs may also benefit from fostering opportunities for men to build meaningful relationships, given our modest but significant findings related to marital status, and incorporating diversion-based to address criminal-legal system involvement. Designing treatment plans that respond to both real-world barriers and clinical needs, especially in ways that reflect the different challenges men and women face, will improve treatment retention and support sustained recovery.

#### Limitations and future directions

This study leveraged a large, nationally representative dataset to explore predictors of treatment outcomes in publicly funded SUD programs. While the dataset provided a substantial amount of information of treatment episodes, it was limited in the specificity of the available variables. Although our dataset captured many treatment episode variables, it lacked important information on psychosocial variables, such as treatment motivation, trauma history, perceived quality of care, quality of social support, and household responsibilities (e.g., childcare) that systematically vary by sex. Their omission may bias our sex-based comparisons of engagement and recovery. Additionally, both substance use at discharge and treatment non-completion outcome variables were recoded using discharge codes rather than long-term behavioral outcomes, limiting insight into sustained recovery. Moreover, the substance use at discharge variable is facility reported and the dataset does not specify whether this is based on client self-report, urine toxicology, or clinical observation. Furthermore, because the dataset only includes individuals who had already been discharged, it excludes those still in treatment and may not reflect the full range of treatment participation or continuity. Several predictors were also recoded into broader categorical variables, which may obscure within-group differences. Future research should incorporate more detailed, longitudinal designs, including post-discharge outcomes and stronger psychosocial data, to better understand recovery patterns and treatment engagement across sexes.

## Conclusion

Findings from this study highlight the need for sex-informed approaches to SUD treatment that address both clinical severity and structural risk factors. Tailoring treatment approaches to reflect these distinct patterns may improve recovery outcomes. As treatment systems continue to evolve, integrating these insights can help close longstanding gaps in care and better support sustained recovery. 

## Data Availability

Data is available upon reasonable request.
